# Retrospective Investigator-Initiated Trial on Tocopherol Acetate Vaginal Administration in Pre-and Postmenopausal Women

**DOI:** 10.3390/diseases12100237

**Published:** 2024-10-02

**Authors:** Noemi Venditti, Giulio Petronio Petronio, Antonio Guarnieri, Laura Pietrangelo, Angela Spicciato, Alessio Colalillo, Giovanna Paola Sabusco, Dionisio Franco Barattini, Aldo Di Franco, Stefano Papini, Francesco Cosentino, Roberto Di Marco

**Affiliations:** 1 Department of Medicina e Scienze della Salute “V. Tiberio”, Università degli Studi del Molise, 86100 Campobasso, Italy; noemi.venditti@responsible.hospital (N.V.); a.guarnieri@studenti.unimol.it (A.G.); laura.pietrangelo@unimol.it (L.P.); angelaspicciato@gmail.com (A.S.); sabusco.giovannapaola@gmail.com (G.P.S.); francesco.cosentino@unimol.it (F.C.); roberto.dimarco@unimol.it (R.D.M.); 2 UO Laboratorio Analisi, Responsible Research Hospital, 86100 Campobasso, Italy; stefano.papini@responsible.hospital; 3 UO Oncology, Responsible Research Hospital, 86100 Campobasso, Italy; alessio.colalillo@studenti.unimol.it; 4 Opera CRO, a Tigermed Company Strada Cozia N. 10, 300209 Timisoara, Romania; barattini@operacro.com; 5 UOC Laboratorio Analisi, Ospedale “A. Cardarelli”, 86100 Campobasso, Italy; aldo.difranco@asrem.org; 6 Department of Drug and Health Sciences, Università degli Studi di Catania, 95100 Catania, Italy

**Keywords:** menopause, vaginal atrophy, genitourinary syndrome (GSM), tocopherol acetate, vaginal microbiota, *Lactobacillus*, vaginal health, eubiosis, vitamin E administration, microbial diversity indices

## Abstract

Background: Menopause, a natural phase in a woman’s life, often adversely affects physical, mental, sexual, and emotional well-being due to low estrogen levels. This study examines the impact of vaginal ovules with tocopherol acetate (Filme Gyno-V^®^ ovules, manufactured by Panin Srl and distributed by Hulka Srl, Italy), 500 mg per ovule, on vaginal health in pre- and menopausal women. Methods: Fifty women aged 50–70 were divided into menopausal (28) and premenopausal (22) cohorts and treated with the ovules for two weeks, with assessments before and after treatment. Results: The findings showed that distressing symptoms of vaginal atrophy, such as dryness, itching, and pain during intercourse, were resolved post-treatment. A molecular analysis revealed a reduction in *Escherichia coli* in both cohorts and an increase in three species of *Lactobacillus* in premenopausal patients. Conclusions: This study concludes that Filme Gyno-V ovules may benefit vaginal health by alleviating atrophy symptoms and promoting healthy vaginal microbiota.

## 1. Introduction

In healthy women of reproductive age, the vaginal microbiota is mainly composed of the genus Lactobacillus bacteria. Although under normal conditions, the vaginal microbiota has a relatively modest biodiversity, and its composition can change during woman’s life cycle [1].

In this scenario, lactobacilli are essential for maintaining vaginal health because they limit the growth of pathogenic bacteria through several direct and indirect ways. These encompass both mechanical and biological mechanisms. The biochemical ones involve the fermentation of glycogen, which produces lactic acid as a byproduct. Lactic acid lowers vaginal pH (3.5–4.5), killing or inactivating vaginal infections. On the other hand, biomechanical ones include the formation of bacterial colonies that adhere to vaginal epithelial cells and create a physical barrier against pathogen adhesion [2]. Like the gut, the vaginal microbiota can also act as a significant modulator of inflammatory responses in the female genitourinary tract. In this regard, a study by Anahtar et al. demonstrated a correlation between alterations in vaginal microbial diversity and increased concentrations of pro-inflammatory cytokine [1].

Recent studies highlight the importance of the vaginal microbiota for the mental and physical health of women at every stage of life. A balanced vaginal microbiota (eubiosis) contributes to the maintenance of an acidic pH, thus preventing infection and protecting the genital tract [1,2].

During infancy, the vaginal habitat tends to have a slightly alkaline or neutral pH, with the microbiota comprising mainly Gram-negative and Gram-positive anaerobic bacteria. During the prepubertal period, thin vaginal mucosa, together with low estrogen and glycogen levels, contribute to the limited lactobacilli presence. Increased estrogen levels during adolescence cause the vaginal epithelium to mature. This increases glycogen deposition in the vaginal epithelium, which promotes the growth of bacteria that produce lactic acid [3].

When estrogen levels drop toward the conclusion of the reproductive period, vaginal epithelial cells retain less glycogen [4], which lowers lactobacilli populations, lowers lactic acid, and raises vaginal pH [5].

Potential pathogens can colonize the vaginal mucosae and change the microbiota when the pH is alkalinized. Low estrogen levels in postmenopausal women also cause anatomical alterations such as decreased blood flow, flexibility, and dryness in the vagina [6,7,8]. During premenopause, the vaginal microbiome plays a key role in influencing reproductive health and outcomes. The presence of *Lactobacillus* species, which produce lactic acid and maintain an acidic vaginal pH, is critical in preventing infection, pelvic inflammatory disease (PID), and sexually transmitted diseases (STDs) [9]. It is also associated with better reproductive health parameters, higher implantation rates in successful pregnancies, and lower rates of miscarriage [10]. In contrast, a microbiota dominated by anaerobic Gram-negative bacteria correlates with low reproductive rates, with reduced pregnancies, and increased risk of recurrent miscarriage [9,10]

However, several factors, such as menstrual cycle, sexual activity, contraceptive use, and lifestyle can influence the high dynamism of the vaginal microbiota with an alteration of the protective *Lactobacillus* species balance, allowing potentially pathogenic bacteria to thrive [9]. This imbalance often made antibiotic and probiotic therapies necessary to correct microbial dysbiosis and improve reproductive outcomes [10]. 

Although a physiological reduction in *Lactobacillus* species is observed with advancing age, these bacteria remain important for maintaining vaginal homeostasis. Recent studies show that postmenopausal women with a lactobacillus-dominant microbiota and lower microbial diversity have lower levels of pro-inflammatory markers such as IL-1b, IL-6, IL-8, and IL-10, suggesting that Lactobacilli continue to exert beneficial effects [11]. In addition, the shift from a *Lactobacillus-*dominated microbiota to a more diverse one, often characterized by an increase in anaerobic bacteria, is linked to urogenital symptoms and infections, especially in postmenopause [12,13]. Physiological menopause is described as the “permanent cessation of menstruation resulting from loss of ovarian follicular activity” by the World Health Organization (WHO) [14]. When amenorrhea persists for 12 consecutive months, the synthesis of progesterone and estrogen declines while follicle-stimulating hormone (FSH) and luteinizing hormone (LH) levels rise, signaling the onset of menopause. It usually affects women between the ages of 49 and 52; in developed countries, the average age is 51.4. However, menopause is mainly influenced by genetic, hormonal, environmental, and lifestyle factors. Indeed, early menopause can arise between the ages of 40 and 44 in women diagnosed with early ovarian failure due to prolonged amenorrhea, hyper-gonadotropinemia, or estrogen deficiency [15].

Symptoms that characterize menopause include hot flashes, night sweats, palpitations, decreased vaginal lubrication, and thinning of the vaginal mucosa, which can cause painful intercourse (dyspareunia), behavioral problems, and other neurophysiological disorders such as mood swings, insomnia, anxiety and depression, decreased ability to concentrate, urinary incontinence, and loss of libido, but may also include paresthesias, nervousness, melancholy, dizziness, weakness, arthralgias, myalgias, and headaches [16].

Hypoestrogenism associated with menopause has a significant negative impact on vaginal and urinary health, often leading to a condition known as genitourinary syndrome (GSM), a term coined by the International Society for the Study of Women’s Sexual Health and the North American Menopause Society in 2014 [17]. GSM is related to genital symptoms such as dryness, burning, irritation, and sexual symptoms such as discomfort and impaired function. Formerly known as vulvovaginal atrophy (VVA), this condition can also manifest with incontinence, dysuria, strangury, and frequent urinary tract infections [18].

All the GSM symptoms can be successfully managed by a variety of products including topical vaginal estrogen, hormone therapy, selective estrogen receptor modulators, long-acting moisturizers, and personal lubricants [19,20,21,22]. In particular, low-dose vaginal estrogen administration has been proven to reach optimal effectiveness with minimal side effects [20]. On the other hand, the estrogen-based approach can represent a limiting factor for cultural reasons, fear of cancer risk increase, and contraindications. For this reason, a recently developed medical device containing tocopherol acetate (Filme Gyno-V^®^ ovules, manufactured by Panin Srl (Rovigo, Italy) and distributed by Hulka Srl, Rovigo, Italy) is widely used by gynecologists in Europe to relieve vaginal dryness and related symptoms. Tocopherol acetate, the synthetic form of vitamin E, offers several benefits to the vaginal mucosa. Vitamin E has phytoestrogenic properties and can alleviate various symptoms of menopause, including those affecting the vaginal mucosa, including vaginal dryness. In addition, vitamin E can serve as an alternative or supplement to hormone therapy, especially in women for whom estrogen therapy is contraindicated or undesirable [23].

Tocopherol acetate also acts on the vaginal mucosa mechanically. As noted by Panin et al. (2004), it creates a protective oily film on the surface to which it is applied, which reduces friction and prevents further trauma. This oily film helps restore and maintain normal moisture balance, minimizing transepidermal water loss, a common problem in menopausal vaginal dryness. In addition, the antioxidant and anti-inflammatory properties of tocopherol acetate, combined with its role in improving skin hydration and repair, promote collagen synthesis and contribute to the overall health of the vaginal mucosa [22].

In addition, it promotes the growth of bacterial species, especially lactobacilli, in the vaginal environment. By promoting microbial activity, it creates a protective, moist environment contributing to maintaining a vaginal physiological pH balance and preventing infection [22,23]. Although this vaginal suppository formulation of tocopherol acetate appears to be widely used in clinical practice and its efficacy for GSM symptoms is recognized by both prescribers and patient users, few trials [24] have evaluated its performance in an outpatient clinical practice settings. The present study aims to fill this gap and analyze the efficacy of the vaginal suppository formulation of tocopherol acetate in decreasing the signs and symptoms of GSM in premenopausal and postmenopausal women. A further aim of this study is to verify whether tocopherol acetate ovules can promote vaginal eubiosis by acting on the lactobacillary flora and its effects on the microbiota. Patients underwent an initial assessment at baseline that included the collection of vaginal swabs, followed by treatment with vaginal tocopherol acetate administration for 14 days. Subsequently, at the end of the treatment period (final visit), the patients underwent another assessment that included the collection of vaginal swabs.

## 2. Materials and Methods

### 2.1. Study Design

This is an observational, open not comparative, monocentric, retrospective Investigator-Initiated Trial (IIT). The comparison was performed using retrospectively collected data belonging to the clinical and microbiological evaluation, performed by the physician before and at the end of the treatment period on patients during the normal course of care. The included patients were premenopausal and menopausal women who visited the UOC Gynaecology and Oncology of the Responsible Research Hospital, Campobasso (Italy), between 1 January 2020 and 31 December 2022, for GSM symptoms and who were treated according to the standard clinical practice of the site with the medical device containing tocopherol acetate in an vaginal suppository formulation. Additional molecular examinations were carried out in a limited part of the involved population, and the data were analyzed at the Laboratory of Clinical Microbiology, Department of Medicine and Health Sciences “V. Tiberio”, University of Molise, Campobasso (Italy).

A formal sample size calculation was not carried out given the nature of the observational study.

### 2.2. Patient Population 

The patients met the following inclusion criteria: female sex; an age of ≥50 to ≤70 years inclusive; pre- and menopausal according to the STRAW criteria [25]; and without genitourinary pathologies and/or anatomical and physiological alterations of the vagina at the time of recruitment. The exclusion criteria were oncological treatment and/or previous radiotherapy/brachytherapy; on the other hand, patients were included even if they had benign gynecological pathologies such as ovarian cysts, uterine myomas/fibroids, and endometriosis; previous physical treatments on the vagina/cervix (i.e., diathermocoagulation/laser); current estroprogestin hormone therapy; antibiotic therapy in the previous 15 days; and/or a known allergy to tocopherol.

Our team, considering the standard annual visit rate of the UOC Gynecology and Oncology, initially planned to collect data from 75 patients (25 menopausal and 50 perimenopausal plus premenopausal) that visited the healthcare facility from 1 January 2020 to 31 December 2022. During the SARS-CoV-2 pandemic, the number of subjects seen for symptoms of GSM in our department at the Responsible Research Hospital decreased dramatically; therefore, we could collect data from only 50 patients. In the present analysis, we divided the population into two cohorts: menopausal (28 women) and premenopausal patients (22 women).

### 2.3. Treatment

Patients who visited our center between 1 January 2020 and 31 December 2022 were prescribed tocopherol acetate ovules daily for 14 days. One ovule (containing tocopherol acetate 500 mg) was self-administered intravaginally every evening before bedtime, as indicated in the relative instructions for use. Moisturizers, personal lubricants, or products used to treat symptoms of vaginal atrophy were not allowed during the study period.

### 2.4. Time Points and Data Collection

The data collected and presented here relate to patients seen in the standard usual care setting at our center; therefore, patients were seen at baseline and on day 14, which are the intervals provided in our normal clinical routine for GSM symptoms, regardless of their participation in this study.

After receiving approval from the Ethics Committee notification, data were collected and analyzed from 20 April to 20 May 2024.

### 2.5. Clinical and Microbiological Evaluation

The patients underwent a gynecological examination and were interviewed to obtain information about the presence of subjective symptoms of vaginal atrophy (dryness, itching, dyspareunia, and burning). Subsequently, each patient underwent an objective examination of vaginal status according to Tucker et al. [4]. Each clinical sign was classified on a 4-point scale to express severity (0 = none or expected (normal), 1 = mild, 2 = moderate, and 3 = severe). Before (T0) and after (T1) treatment with Filme Gyno-V ovules, the following clinical parameters were assessed:

- Subjective symptoms of vaginal atrophy (dryness, itching, dyspareunia, and burning);
- Objective examination of the vaginal mucosa status (secretions, color, integrity, and thickness).

Vaginal secretions gathered from the lateral wall of the upper third of the vagina were tested for pH using a pH indicator tape and KOH production via a sniff test.

Furthermore, the collected material was fixed on a slide for Gram staining and analyzed under a light microscope for assessment of *Lactobacillus* grade according to the Ison–Hay scale (validation of a simplified grading of Gram-stained vaginal smears for use in genitourinary medicine clinics)

Culture examination and detection of aerobic bacteria, anaerobic bacteria, and pathogenic yeasts were performed on selective media: Columbia Agar + 5% sheep blood with nalidixic acid and colistin (CNA), Gardnerella agar (GAR), Chocolate agar PolyViteX (PVX), Schaedler agar + 5% sheep blood (SCS), Chocolate agar PolyViteX VCAT3 (VCA3) (Biomerieux, Paris, France), and Candida Bromcresol Green (BCG) Agar (Vacutest Kima S.r.l, Padova, Italy). Fenotipical identification was conducted by Vitek Two (Biomerieux, Paris, France).

### 2.6. Molecular Analysis

#### 2.6.1. Population Selection

Genomic sequencing analyses were conducted on a portion of the population involved in the clinical trial to optimize the use of financial resources and reduce analysis time. Out of 50 patients (40%), 20 underwent molecular analysis, with 13 belonging to the premenopausal cohort and 7 to the menopausal cohort. The selection of the population subset for molecular analyses was determined by assessing the results of clinical and microbiological evaluations.

#### 2.6.2. Sampling

Vaginal microbiota sampling was conducted by the gynecologists in charge using e-nat swabs (Copan eNat^®^, COPAN ITALIA spa, Brescia, Italy), as recommended by the manufacturer’s procedure, in sterile conditions, applying firm pressure and vigorously rubbing the swab back and forth 50 times (for 30 s). This process was conducted both before and after treatment with Filme Gyno-V ovules. Subsequently, the collected vaginal microbiota samples were transported to the laboratory under controlled temperature conditions of 4 °C and stored at −80 °C until further processing.

#### 2.6.3. Procedure

The T0 and T1 swabs were processed at BioRep s.r.l. (Milan, Italy). Firstly, the extraction of DNA was performed using the QIAamp DNA Microbiome kit © (QIAGEN, Hilden, Germany). Moreover, the DNA isolated was sequenced through the Shotgun DNA-Seq approach (20 M reads). The Shotgun DNA-Seq raw data underwent analysis using Gaia software V. 2.0 (Sequentia Biotech s.r.l.). Forward and reverse FASTQ files were initially decompressed and subjected to sequencing quality evaluation (Quality Control, QC). Subsequently, the trimmed reads were merged into overlapping paired-end reads, and only the high-quality reads were retained. The full-length Shotgun genomes were concurrently matched against the WGS and WTS Prokaryotes (bacteria and archaea), WGS and WTS Fungi, and WGS and WTS Viruses databases (all released in 2020), customizing NCBI genome sequences to the reference databases. The Operational Taxonomic Units (OTUs) were established based on at least 97% identity, operating at the species level. A taxonomy table describing the abundance of every OTU in each microbiota was created by cross-referencing the OTU table with the taxonomy entries. This taxonomy table was used for statistical analysis and gave a microbiota profile for all samples.

Given the potential impact of sample heterogeneity (premenopausal and postmenopausal) on result significance, a multi-step data analysis was undertaken for the Shotgun sequences. Initially, the vaginal microbiotas of the T0 and T1 groups were compared to identify common variations across all samples resulting from treatment with Filme Gyno-V ovules. The vaginal microbiotas of T0 and T1 were then compared within the two groups (premenopausal and menopausal) to find significant dependent differences.

#### 2.6.4. Statistical Analysis

Chi-square tests were used to find patterns and correlations for nominal variables. Non-parametric tests, the Mann–Whitney U test, and parametric techniques like *t*-tests and analyses of variance (ANOVA) were used to investigate correlations for numerical variables. IBM SPSS Statistics v.26 was the statistical program used for these analyses.

The raw OTU table was used to calculate the alpha and beta diversity indices without sample rarefaction. The Shannon index measured evenness in the alpha diversity assessment, while the Chao1 index assessed sample richness. The normality of index distributions was evaluated via the Shapiro–Wilk test (α = 0.05), with statistically normally distributed series compared using Welch’s *t*-test and non-normally distributed series assessed through the Kolmogorov–Smirnov test, both at a significance threshold of *p* ≤ 0.05. The alpha diversity indexes of T0 and T1 vaginal microbiotas were compared across all taxonomic levels. Similarly, being sensitive to taxa abundances, Bray–Curtis distances were computed to appraise sample diversity (beta diversity). All detected domains’ pre- and post-treatment abundances were statistically compared using Welch’s *t*-test for normally distributed series and the Kolmogorov–Smirnov test for non-normally distributed ones (*p* ≤ 0.05).

A further analysis identified the most prevalent taxa at the upper (phylum and class) and lower (genus and species) taxonomic ranks, comparing their abundances in T0 and T1 microbiotas in the premenopausal and postmenopausal groups. The statistical significance of the abundance distribution comparisons was assessed using Student’s *t*-test and the Wilcoxon test (*p* ≤ 0.05 for both) for normal and non-normal distribution of percentages, respectively. Finally, DESeq2 differential analysis was performed to identify potentially differentially represented OTUs between treated (T1) and untreated (T0) vaginal microbiotas.

## 3. Results

### 3.1. Population

The patients underwent a questionnaire upon enrollment to encode general, medical, and study-relevant information. Table 1 shows the collected data.

Out of 50 enrolled patients (mean age 55.00 ± 7.66), 22 were premenopausal (mean age 49.00 ± 3.53), and 28 were menopausal (mean age 59.86 ± 6.41). Six premenopausal and fourteen menopausal patients were receiving therapy in the 14 days before the start of the clinical trial, and one premenopausal patient and six menopausal patients had prior benign diseases.

Twenty-one of the twenty-two premenopausal patients were sexually active, and seventeen of them had given birth at least once: ten via natural means, six via cesarean, and one via both. In contrast, among the 28 menopausal patients, 17 were sexually active, and 25 had completed at least one pregnancy, 14 of which were natural births, 9 cesarean, and 2 both.

The use of contraceptives and potential risk factors such as smoking and body mass index (BMI) were also assessed. Out of the 50 enrolled patients, 1 premenopausal and 2 menopausal patients had used contraceptives at least 10 years before this study began, 6 (2 premenopausal and 1 menopausal) had a BMI ≥ 30 (overweight), and only 1 premenopausal patient was a smoker (Table 1).

### 3.2. Clinical Evaluation

A total of 50 patients were enrolled: 22 in premenopause (PMP) and 28 in menopause (MP). Among these, 33 women reported symptoms of vaginal atrophy (66%), 12 PMP and 21 MP. The assessment of vaginal atrophy symptoms (itching, burning, dryness, and dyspareunia) reported by patients during visits before and after treatment with Filme Gyno-V showed an overall improvement in all the stated symptoms (Table 2).

Before treatment (T0), 66% of the patients reported at least one of the symptoms of vaginal atrophy. After treatment (T1), there was a total resolution of reported symptoms for both study groups, at a rate of 100%, and no worsening in the absence of symptoms. The most prevalent symptom among the PMP patients was dryness (54%), followed by dyspareunia (16%). On the other hand, among the MP patients, dryness ranked highest at 75%, followed by a combination of dryness and dyspareunia at 14%. 

Notably, there were cases when symptoms, including itching or dryness with dyspareunia, overlapped, suggesting that patients’ symptoms may co-occur. Nevertheless, these symptoms also went away after treatment. Therefore, neither the MP nor the PMP groups had any atrophy symptoms.

No observed adverse effect was reported for all treated patients.

Table 3 reports the clinical assessment before (T0) and after treatment with Filme Gyno-V (T1), using the score proposed by Tucker *et al.* [4] on variations in the following parameters:

- Secretions;
- Mucosa color;
- Mucosa integrity;
- Mucosa thickness.

Regarding vaginal secretions, despite a trend reduction observed, there was no statistically significant difference in the prevalence of vaginal secretions between PMP, MP, or total population from T0 to T1.

On the other hand, vaginal color in the MP and total populations demonstrated a statistically significant difference from T0 to T1 (*p* 0.002 and 0.003, respectively). The expected score (normal) had a 183% increase in the MP group and 52% in the total population. In comparison, moderate (pale pink) had a percentage reduction of 90% and 92%, followed by mild (light pink) with 17% and 7% reductions, respectively. Similar findings have been reported for integrity and thickness (all *p*-values are <0.05). The expected score increased for integrity and thickness (50%, 29%; and 111%, 41%) along with a complete resolution of severe vaginal thickness in the MP and total populations (Table 3).

Further parameters such as vaginal pH, the presence of amines (evaluated via the sniff test), and microscopic assessment of polymorphonuclear leukocytes (PMNs) as an indicator of inflammation were also examined.

Regarding pH, no significant variances were found across the entire population between the group at T0 (baseline visit) and the post-treatment group (follow-up). All groups had the same mean pH value both before (T0) and after administration (follow-up) (Table 4). Overall, 14 patients (seven in each group) had a pH drop, 17 patients (15 menopausal and 2 premenopausal) had a little increase in alkalinity, and the remaining 19 patients (13 premenopausal and 6 menopausal) had no change in alkalinity (Table 5).

Table 6 illustrates the assessment of amine presence through the sniff test and PMN score variation. Despite a reduction trend observed in the MP and total population, no significant variation has been observed in all the patients’ groups (Table 6).

### 3.3. Microbiological Analysis

Table 7 lists the isolated microorganisms and their distribution as a monomicrobial or polymicrobial isolation at T0 and T1 for both study groups and the total population. No statistically significant differences were observed between the groups or the two periods under examination.

### 3.4. Ison–Hay Score

The Ison–Hay Score, a measure of bacterial composition in vaginal samples, revealed interesting trends when comparing the PMP, MP, and total populations before (T0) and after (T1) treatment (Table 8).

Highly significant results were found for the PMP group (*p* = 0.007) and the total population (*p* = 0.001), while for the MP group, although an improvement trend was observed, no significant results were recorded.

Initially, at T0, the PMP (18%) and total populations (34%) exhibited no bacterial presence. However, by T1, this percentage decreased significantly to 9% for PMP and 20% for the total population, indicating a shift in bacterial colonization. Moving to Grade 1, characterized by the dominance of Lactobacillus species, there was a slight uptick in both groups from T0 to T1. For PMP, the percentage increased from 32% to 36%, while for the total population, it rose from 18% to 34%.

In Grade 2, where Lactobacilli and Gram-positive cocci co-exist, there was a notable increase from T0 to T1 for both cohorts. PMP saw an increase from 14% to 55%, while the total population increased from 16% to 40%. Moving on to Grade 3, characterized by an overgrowth of Gram-positive cocci and other morphotypes, there was a decrease from T0 to T1 for both groups.

Grade 4, representing the presence of Gram-positive cocci, showed minimal presence overall, with a decrease from T0 to T1 in the PMP group.

Regarding score variation, 39% of the PMP group, 32% of the MP group, and 36% of the total population showed no variation between T0 and T1. Most women, constituting 54% of all cohorts, exhibited improvement over the same period. Conversely, a smaller percentage experienced worsening, with 7% of the PMP group, 14% of the PMP group, and 10% of the total population showing this trend (Table 8).

In summary, the Ison–Hay Score highlights notable changes in bacterial composition and score variation over time in all cohorts, with significant findings observed in specific categories.

### 3.5. Molecular Analysis

#### 3.5.1. Population Selection

Twenty out of fifty patients (40%) underwent molecular analysis, with thirteen in the PM and seven in the PMP cohorts. Table 9 illustrates changes in clinical parameters (atrophy, secretions, color, integrity, thickness, sniff test amines, pH, and PMNs) and microbiological parameters (Ison–Hay Score) post-treatment, categorized into three groups: No Variation (NV), Improvement (IMP), and Worsening (WR).

Of the PMP patients, five had NV, eight exhibited improvement, and none showed worsening of vaginal atrophy symptoms. Five MP patients showed improvement, two showed NV, and none showed worsening. Seven had NV overall, thirteen had improved, and none had worsened.

When it comes to vaginal secretions, ten PMP patients had NV, three had improved, and none had gotten worse. Six MP patients exhibited NV. One showed improvement, and none showed worsening. Overall, four showed improvement, none worsened, and sixteen showed NV.

Eleven PMP patients had NV in terms of vaginal mucosa color, two exhibited improvement, and none worsened. Three MP patients exhibited NV, four showed improvement, and one showed worsening. In the total cohort, fourteen had NV, six had improved, and one had worsened.

Eleven PMP patients demonstrated NV, two exhibited improvement, and none showed worsening of the vaginal mucosa integrity. Six MP patients exhibited NV; one showed improvement, and none showed worsening. Seventeen had NV overall, three had improved, and none had worsened.

Eleven PMP patients had NV in terms of vaginal mucosa thickness, two exhibited improvement, and none worsened. Four MP patients showed improvement, three showed NV, and none showed worsening. All in all, fourteen had NV, six had improved, and none had worsened.

Seven PMP patients showed NV on the sniff test, five exhibited improvement, and one showed worsening. Four MP patients had NV, one exhibited improvement, and two showed worsening. In the total population, eleven had NV, six had improved, and three had worsened.

Regarding pH levels, nine PMP patients showed NV, three showed improvement, and one showed worsening. Two MP patients exhibited NV, three showed improvement, and two showed worsening. Eleven people had NV overall, six of them got better, and three got worse.

Additionally, of the PMP patients, seven had NV for PMNs, four had improvement, and two had worsening. Of the MP patients, four exhibited improvement, none worsened, and three displayed NV. Ten had NV overall, eight had improved, and two had worsened.

Finally, two PMP patients demonstrated NV on the Ison–Hay Score, ten showed improvement, and one exhibited worsening. Of the MP patients, none worsened, seven improved, and none had NV. In the total cohort, two had NV, seventeen had improved, and one had become worse.

#### 3.5.2. Shotgun Sequence Analysis

**Figure 1:**
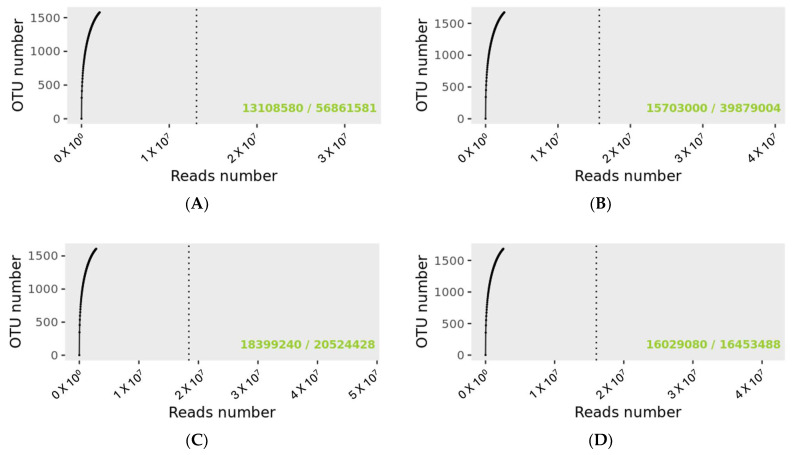
Rarefaction curves for sample size evaluation. (**A**): PMP at T0; (**B**): PMP at T; (**C**): MP at T0; (**D**): MP at T1. 56,861,581 OTUs were detected for the PMP T0 group and 39,879,004 for PMP at T1. For the MP groups, the detected reads were 20,524,428 and 16,453,488 for T0 and T1, respectively.

The rarefaction curve revealing the number of identified OTUs as a function of the read number for MP and PMP groups at T0 and T1 is shown in Figure 1. The dashed vertical line indicates the number of readings needed to observe 95% of the total richness of the PMP and MP groups at T0 and T1. The total number of readings that groups all samples for the specified condition is indicated in the lower right of the graph, just after the bar sign. The number of readings needed to reach 95 percent richness is indicated before the bar mark. The green text indicates that the number of reads needed to observe 95% richness is sufficient.

Matching with the Gaia WGS and WTS databases for prokaryotes, fungi, and viruses identified a total of 56,862,699 OTUs for the PMP group at T0 with a mean value per sample of 4,374,054 and 39,880,029 at T1 with a mean value per sample of 3,067,695 matchings. As regards the MP group at T0, there was a total of 20,525,403 and 16,454,315 OTUs at T1 with a mean value per sample of 2,565,675 at T0 and 2,056,789 at T1.

Although there were different numerosities among the sample groups, no significant difference was found for the number of corresponding OTUs (Wilcoxon test, PMP *p* = 0.545, and MP *p* = 0.195).

The PMP and MP microbiotas’ qualitative composition at the domain level and at T0 were dominated by the bacteria component (85% and 53%, respectively). After treatment (T1), despite a percentage shift (60% and 68%, respectively), the bacteria domain remained prevalent.

Subsequently, matching with the Gaia WGS and WTS databases for prokaryotes alone, a total of 52,865,313 OTUs were found for the PMP group at T0 with a mean value per sample of 4,066,562 and 36,280,735 at T1 with a mean value per sample of 2,790,826 matchings. As regards the MP group at T0, a total of 17,310,503 and 13,559,644 OTUs were found at T1 with a mean value per sample of 2,163,813 at T0 and 1,694,955 at T1.

Although the sample groups had different numerosities, no significant difference was found for the number of corresponding OTUs (Wilcoxon test, PMP *p* = 0.687, and MP *p* = 0.208).

The alpha diversities of PMP and MP prokaryotes before (T0) and after treatment (T1) were evaluated at all taxonomic levels. No statistical differences were found in prokaryotes’ alpha and beta diversities in both study groups (Supplementary Materials, Table S1).

The beta diversity of the PMP and MP prokaryotes before (T0) and after treatment (T1) were also evaluated at species levels by a distance matrix representing Bray–Curtis dissimilarity values (Supplementary Materials, Table S2). For the PMP cohort, out of the thirteen patients analyzed, only two reported dissimilarity values close to 1, specifically 0.857 and 0.665 (Supplementary Materials, Table S2A), while for the MP cohort, only one patient showed dissimilarity before and after treatment (0.722) (Supplementary Materials, Table S2B).

After the statistical analysis of the diversity indices, a differential DESeq2 analysis was conducted to identify the statistically differential OTUs in the two groups under study, T0 and T1.

**Figure 2:**
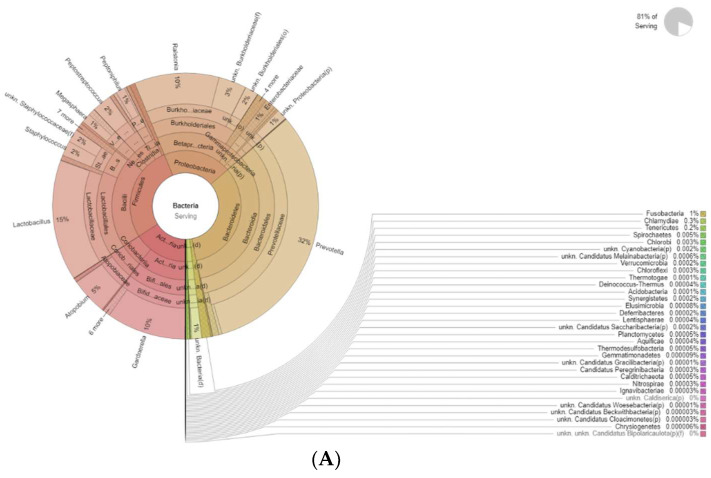

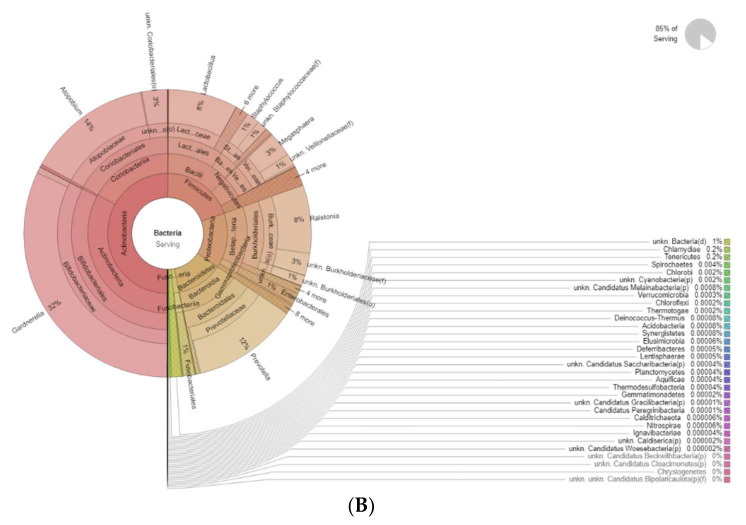
Krona plots showing differences in bacteria genera belonging to the vaginal microbiota. (**A**) PMP before treatment (T0), (**B**) PMP after treatment (T1).

The differences in bacteria genera belonging to the vaginal microbiota in the PMP group before (T0) and after treatment (T1) are reported in Figure 2. 

The results of a differential abundance analysis comparing microbial communities at species level are expressed as log fold change (logFC) for each OUT in the PMP cohort (T1 vs. T0). 

**Figure 3:**
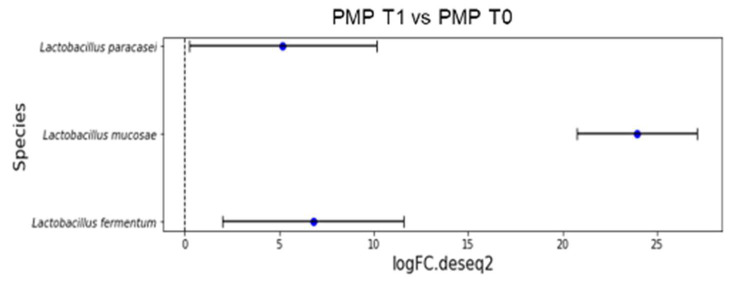
Overrepresented species from differential abundance analysis comparing microbial communities at species level expressed as log fold change (logFC) for each OUT in PMP cohort (T1 vs. T0).

Figure 3 reports the overrepresented species after treatment (*p* < 0.001):

- *L. fermentum* (LogFC = 6.78);
- *L. mucosae* (LogFC = 23.96);
- *L. paracasei* (LogFC = 5.19).

**Figure 4:**
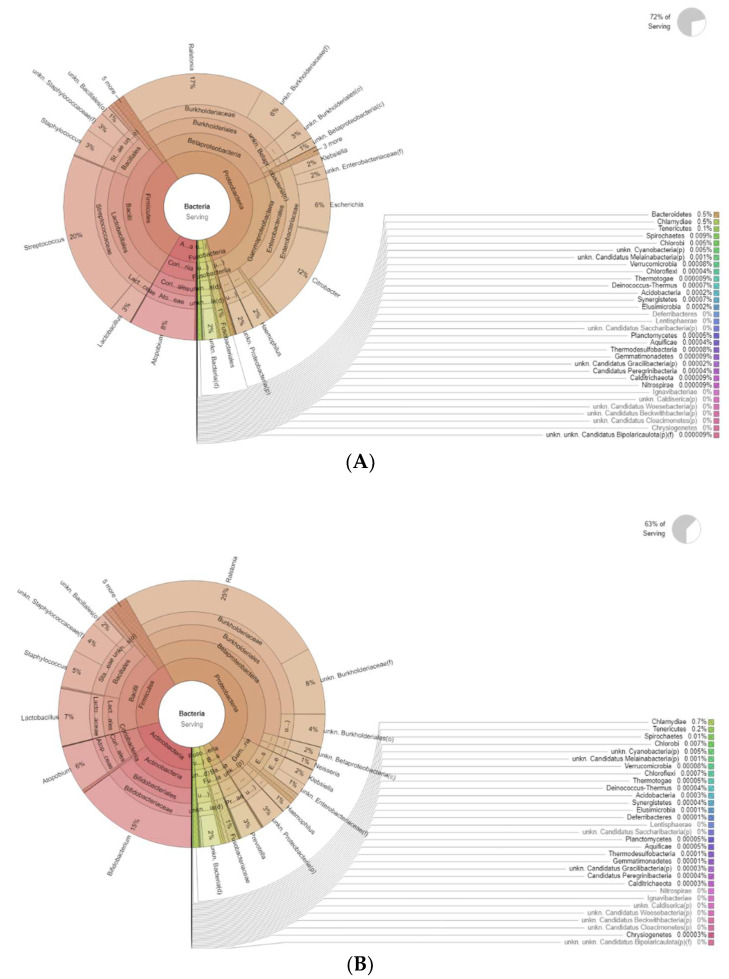
Krona plots showing differences in bacteria genera belonging to the vaginal microbiota. (**A**) MP before treatment (T0), (**B**) MP after treatment (T1).

Figure 4 shows the differences in bacteria genera belonging to the vaginal microbiota in the MP group before (T0) and after treatment (T1). The results of a differential abundance analysis comparing microbial communities at the species level are expressed as log fold change (logFC) between the two groups for each OTU. 

The results of the differential DESeq2 analysis, T1 vs. T0, on OTUs belonging to biogenic amine (BA) producers’ bacterial genera in the two groups under study are reported in Table 10 and Table 11. OTU changes are given as log fold change (logFC).

In the PMP group (Table 10), *Mobiluncus* spp. does not show a statistically significant change, with a negative logFC of −1.608 and a *p*-value of 0.161. On the other hand, an increase is observed in the MP group (logFC of 4.666, *p*-value of 0.003) (Table 11). 

For *Peptostreptococcus* spp. in the MP group (Table 11), there is an increase close to statistical significance (logFC = 2.616; *p* = 0.031). In contrast, in the PMP group (Table 10), a significant decrease (logFC = −2.915; *p* = 0.010) was observed. The other bacterial-genera BA producers, such as *Klebsiella* spp., *Parvimonas* spp., *Prevotella* spp., *Enterobacter* spp., *Gardnerella* spp., *Clostridium* spp., *Citrobacter* spp., *Megasphaera* spp., and *Leptotrichia* spp., showed no statistically significant changes between T0 and T1 in either group.

Table 11 shows a significant reduction in *E. coli* abundance in the MP group at time T1 compared to time T0, with a logFC of −6.78 and a *p*-value of less than 0.001. Similar behavior is also observed in the PMP group, where *E. coli* undergoes a significant reduction (logFC = −2.02; *p* < 0.001) (Table 10). 

## 4. Discussion

According to this study’s findings, premenopausal and menopausal women’s vaginal use of Filme Gyno-V ovules containing tocopherol acetate had encouraging results in terms of improving many vaginal health-related indicators. Symptoms of vaginal atrophy, such as burning, itching, dryness, and dyspareunia, significantly improved. Notably, following treatment, all reported symptoms wholly resolved in both the premenopausal and menopausal groups.

In addition, the clinical assessment demonstrated favorable improvements in vaginal mucosa features like color, integrity, and thickness after administration. Moreover, the Ison–Hay Score results showed a noteworthy improvement in the microscopic appearance of Gram-stained vaginal smears, suggesting a transition towards a healthier vaginal microbiota profile. 

The antioxidant and preventive qualities of vitamin E may be responsible for these improvements, which point to a restoration of vaginal eubiosis. The observed outcomes align with previous research indicating the beneficial effects of vitamin E on vaginal health. In this scenario, in 2022, Feduniw et al. conducted a systematic review of 83 research studies finding out that the administration of vitamin E help to reduce postmenopausal symptoms such as hot flashes, vascular control, blood lipid profile, and vaginal alterations [23]. Furthermore, in 2016, Parnan Emamverdikhan et al. investigated the therapeutic effects of vitamin E on vaginal atrophy, underlining its significance. During a 12-week administration period, vitamin E suppositories substantially benefited vaginal atrophy management and promoted vaginal maturation value (VMS) [26].

As far as we know, this is the first scientific manuscript that analyzes the bacterial composition of the vaginal microbiota in premenopausal and menopausal women after the administration of vitamin E. Previous research on orally administered tocopherol conducted by Ataei-Almanghadim et al. (2020) found that orally taken vitamin E significantly reduced the frequency of hot flashes but did not improve other symptoms, such as anxiety, sexual function, or general well-being during the menopausal period [27]. In contrast, our study and other studies examining the use of vitamin E, administered through vaginal suppositories, showed different results. Ziagham et al. compared the effects of vaginal suppositories of vitamin E and hyaluronic acid on atrophic vaginitis. Although both treatments significantly relieved symptoms and improved vaginal pH and epithelial maturation, hyaluronic acid provided superior relief. However, vitamin E was effective in reducing vaginal pH and improving epithelial maturation, indicating its potential as a treatment option, especially for women who cannot or prefer not to use estrogen-based therapies [28,29]. Given that genomic sequencing can be costly and time-consuming, it was decided that we would focus these analyses on a representative subgroup of the study population. This approach aimed to maximize the scientific value of the genomic analyses by concentrating our efforts on those participants most likely to benefit from the genetic information gathered. Such an approach allowed us to balance the depth of analysis and resource availability while enabling a comprehensive and exhaustive evaluation of the clinical trial outcomes. 

The same operator collected and managed the microbiota swabs from the same laboratory for genomic extraction and sequencing. Indeed, as reported in the rarefaction curve analysis (Figure 1), the number of reads needed to observe 95% for all the four sample groups’ (PMP and MP at T0 and T1) richness was sufficient, confirming the adequate standardization of sampling procedures and genomic extraction.

The microbiota raw data were managed through Gaia software, V. 2.0 (Sequentia Biotech srl, Barcellona, Spain) using a two-step matching approach. In the first step, a match with WGS and WTS databases for prokaryotes, fungi, and viruses showed the predominance of the bacteria domain in both study cohorts. In the second step, a subsequent match with the prokaryote database allowed us to deepen, at all domain levels, the differences between the before (T0)- and after-treatment (T1) microbiota for the PMP and PM groups.

All the analyses were conducted at both quantitative and qualitative levels, encompassing evaluations of alpha and beta diversities of the identified microbial communities and the detection of significant (DESeq2 analysis) and noteworthy variation trends among the observed OTUs.

Although there were no statistical shifts in the alpha diversity observed in both the PMP and MP cohorts after treatment (as outlined in the Supplementary Materials, Table S1), the positive impacts of vitamin E on the vaginal microbiota might not be attributable to these particular variables, even if there were no changes in the alpha diversity. On the other hand, the beta diversity results showed that out of the 13 PMP patients before and after treatment, 2 had dissimilarity values of 0.850 and 0.66; therefore, these two samples exhibited significant changes in microbial community composition between the pre-treatment and post-treatment periods (Supplementary Materials, Table S2A). Similar considerations can be made for the MP cohort, where only one out of seven patients showed a beta diversity value of 0.772 (Supplementary Materials, Table S2B)

Numerous scientific studies have provided evidence to corroborate the link between menopause and composition of the vaginal microbiome changes. 

Indeed, in a previous study from Moosa et al. higher levels of vaginal E. coli were found, along with an increased frequency of coliform and Gram-negative E. coli when comparing menopausal to premenopausal women [30].

Moreover, BAs play an important role in vaginal homeostasis. BAs are organic compounds produced through the decarboxylation of amino acids by bacteria. They are biochemical indicators of microbial metabolism that occur in environments where significant changes in pH are observed [31]. Vaginal pH influences, and is influenced by, the growth and activity of microorganisms. Under physiological conditions, it settles at around 3.8 and 4.5 [32]. However, when the pH shifts to more alkaline values, this can facilitate the proliferation of anaerobic bacteria, which possess the ability to produce BAs such as putrescine, cadaverine, and trimethylamine through the activation of specific metabolic pathways, such as the decarboxylation of ornithine and lysine. This process is self-regulating, as the very BAs themselves contribute to further raising the pH, creating positive feedback that amplifies the imbalance [32]. 

Thus, the variables affecting the overall composition of the vaginal microbiota are complex and reflect the interplay between host effects on the vaginal environment and interactions between bacterial communities. Thus, a comprehensive grasp of both host-related and microorganism-related mechanisms is crucial for devising effective interventions.

All these findings have been confirmed by DEseq2 analysis. After treatment for both groups, the underrepresented species was *E. coli*., −2.02 LogFC for PMP and −6.78 LogFC in PM (*p* < 0.001). On the other hand, as reported in Table 9 and Table 10, other BA-producing coliforms (*Klebsiella* spp., *Enterobacter* spp., and *Citrobacter* spp.) show no statistically significant changes after treatment, as is the case with other genera such as *Gardnerella* spp. and *Clostridium* spp.

Furthermore, in the group PMP, a decrease in *Peptostreptococcus* spp. was reported, while a slight increase (*p* = 0.031) was reported for MP. In the MP group only, an increase in *Mobiluncus* spp. was observed after treatment. This evidence finds a possible explanation in a 2015 paper by Nelson et al. In this manuscript, the authors demonstrated that only certain bacterial taxa responding positively to pH increase, including *Peptostreptococcus* and *Mobiluncus* spp., are abounding in vaginal microbiota with reduced presence of *Lactobacillus* spp. [32].

Indeed, three different species of *Lactobacillus* were overrepresented after treatment (*p* < 0.001): *L. fermentum* (LogFC = 6.78), *L. mucosae* (LogFC = 23.96), and *L. paracasei* (LogFC = 5.19) in the PMP cohort.

*L. fermentum*, one of the predominant *Lactobacillus* spp. in healthy women’s gut and vaginas, can restore or maintain eubiosis and shield against infections. Synthesizing substances like H_2_O_2_, bacteriocin, and biosurfactants inhibit the pathogen’s growth of pathogens, improving bacterial biodiversity by lowering the number of pathogens. Furthermore, the probiotic activity of *L. fermentum* has been shown against Gram-negative bacteria, such as *S. typhimurium*, *E. coli*, and *K. pneumoniae* biofilms [33,34]. 

Comparably, *L. paracasei* bacteriocins have demonstrated antibacterial action against several pathogens, including *E. coli*, *p. aeruginosa*, *S. aureus*, *S. pyogenes*, and *S.epidermidis.* Furthermore, these bacteriocins remained stable under various factors, such as heat treatment and pH alkalization [35]. 

Lastly, *L. mucosae* is a well-known colonizer in the human vagina as well as the animal gut.

As regards its probiotic activity, genomic studies have identified genes that encode postbiotic compounds such as α- and β-galactosidases, β-glucuronidases, β-fructosidases, and α-amylases, along with a neopullulanase enzyme that can break down pullulan to produce panose, which is utilized by beneficial bacteria. Furthermore, *L. mucosae* can be a natural barrier for protecting epithelial cells [36].

These data suggest that Filme Gyno-V likely exerts a negative modulatory effect against *E. coli* in both PMP and MP cohorts and promotes the *Lactobacillus* spp. in the PMP group.

## 5. Conclusions

To sum up, the encouraging results of intravaginal application of Filme GynoV ovules containing tocopherol acetate in premenopausal and menopausal women underscore the potential advantages of vitamin E administration in enhancing vaginal health parameters. In particular, this study showed promise in improving genitourinary health outcomes by reducing symptoms linked to vaginal atrophy and positively modulating the vaginal microbiota by promoting its eubiosis. 

To completely understand the underlying mechanisms of action and evaluate the long-term effects of vitamin E intake on vaginal health, more research is necessary. Research is still needed to fully comprehend the effects of Filme Gyno-V ovules with tocopherol acetate on vaginal microbiota and genitourinary health. However, for premenopausal and menopausal women suffering from vaginal atrophy and its associated symptoms, it is an effective therapeutic intervention.

## Figures and Tables

**Table 1:** A description of the general and medical characteristics of the study population.

	Premenopausal n = 22	Menopausal n = 28	Total n = 50
Age			
Mean ± SD	49.00 ± 3.53	59.86 ± 6.41	55.00 ± 7.66
Median	49	58.5	54
Previous benign pathology			
Metrorrhagia	0	1	1
Endometrial thickening	0	1	1
Endometrial polyp	1	2	3
Cervical polyp	0	2	2
Ongoing therapy or within the last 15 days			
Antimicrobials	0	0	0
Others *	3	12	15
Oral supplements and/or probiotics	3	2	5
Sexual activity			
Present	21	17	38
Absent	1	11	12
Pregnancy: type of delivery			
Absent	5	3	8
Present	17	25	42
Natural birth	10	14	24
Cesarean	6	9	15
Natural and cesarean	1	2	3
Risk factors			
Smoking	0	1	1
BMI ≥ 30	2	4	6
Smoking and BMI ≥ 30	1	0	1
Contraceptives			
Present (in the last 10 years)	1	2	3
Absent	21	26	47

* Others: antihypertensives, hypoglycemic agents, anxiolytics, thyroid hormones, antithrombotics, GnRH analogs, aromatase inhibitors, antiplatelet, osteoporosis treatment.

**Table 2:** Number of patients reporting symptoms of vaginal atrophy at T0 and T1. Percentages are expressed in parentheses. *p* < 0.05.

	Premenopausal (PMP) n = 22 (%)		Menopausal (MP) n = 28 (%)		Total n = 50 (%)	
	T0	T1	T0	T1	T0	T1
**Symptoms of Atrophy**	12 (54)	0 (0)	21 (75)	0 (0)	33 (66)	0 (0)
Dryness	7 (17)	0 (0)	11 (52)	0 (0)	18 (54)	0 (0)
Itching	1 (8)	0 (0)	0 (0)	0 (0)	1 (3)	0 (0)
Dyspareunia	2(16)	0 (0)	0 (0)	0 (0)	2 (6)	0 (0)
Malodorous discharge	0 (0)	0 (0)	1 (5)	0 (0)	1 (3)	0 (0)
Dryness and dyspareunia	2 (16)	0 (0)	3 (14)	0 (0)	5 (15)	0 (0)
Dryness and itching	0 (0)	0 (0)	1 (5)	0 (0)	1 (3)	0 (0)
Itching and burning sensation	0 (0)	0 (0)	2 (9)	0 (0)	2 (6)	0 (0)
Dryness, itching, and burning sensation	0 (0)	0 (0)	1 (5)	0 (0)	1 (3)	0 (0)
Dryness, dyspareunia, and burning sensation	0 (0)	0 (0)	2 (9)	0 (0)	2 (6)	0 (0)
**No Symptoms of Atrophy**	10 (46)	10 (46)	7 (25)	7 (25)	17 (33)	17 (33)

**Table 3:** Patients’ clinical signs at T0 and T. ∆% percentage change, T1 vs. T0. Percentages are enclosed in parentheses, and statistically significant data (*p* < 0.05) are in bold.

Score	**Vaginal secretions**												
		**Premenopausal** **(PMP)** **n = 22 (%)**				**Menopausal ** **(MP)** **n = 28 (%)**				**Total** **n = 50 (%)**			
		T0	T1	Δ%	*p*	T0	T1	Δ%	*p*	T0	T1	Δ%	*p*
	**Expected** (Normal)	18(82)	21(95)	17	0.328	18(64)	25(89)	39	0.162	36(72)	46(92)	28	0.68
	**Mild** (Surface coverage)	3(14)	1(5)	−67		3(11)	1(4)	−67		6(12)	2(4)	−67	
	**Moderate** (Low)	1(5)	0(0)	−100		5(18)	1(4)	−80		6(12)	1(2)	−83	
	**Severe** (None)	0(0)	0(0)	0		2(7)	1(4)	−50		2(4)	1(2)	−50	
Score	**Vaginal color**												
		**Premenopausal ** **(PMP)** **n = 22 (%)**				**Menopausal ** **(MP)** **n = 28 (%)**				**Total** **n = 50 (%)**			
		T0	T1	Δ%	*p*	T0	T1	Δ%	*p*	T0	T1	Δ%	*p*
	**Expected** (Normal)	17(77)	18(82)	6	0.338	6(21)	17(61)	**183**	**0.002**	23(46)	35(70)	**52**	**0.003**
	**Mild** (Light pink)	3(14)	4(18)	33		12(43)	10(36)	**−17**		15(30)	14(28)	**−7**	
	**Moderate** (Pale pink)	2(9)	0(0)	−100		10(36)	1(4)	**−90**		12(24)	1(2)	**−92**	
	**Severe** (Transparent)	0(0)	0(0)	0		0(0)	0(0)	**0**		0(0)	0(0)	**0**	
Score	**Vaginal integrity**												
		**Premenopausal ** **(PMP)** **n = 22 (%)**				**Menopausal ** **(MP)** **n = 28 (%)**				**Total** **n = 50 (%)**			
		T0	T1	Δ%	*p*	T0	T1	Δ%	*p*	T0	T1	Δ%	*p*
	**Expected ** (Normal)	20(91)	22(100)	10	0.148	18(64)	27(96)	**50**	**0.009**	38(76)	49(98)	**29**	**0.004**
	**Mild** (Superficial bleeding upon scraping)	2(9)	0(0)	−100		5(18)	1(4)	**−80**		7(14)	1(2)	**−86**	
	**Moderate** (Superficial bleeding upon light contact)	0(0)	0(0)	0		5(18)	0(0)	**−100**		5(10)	0(0)	**−100**	
	**Severe** (Pre-existing petechiae, bleeding upon contact)	0(0)	0(0)	0		0(0)	0(0)	**0**		0(0)	0(0)	**(0)**	
Score	**Vaginal thickness**												
		**Premenopausal ** **(PMP)** **n = 22 (%)**				**Menopausal ** **(MP)** **n = 28 (%)**				**Total** **n = 50 (%)**			
		T0	T1	Δ%	*p*	T0	T1	Δ%	*p*	T0	T1	Δ%	*p*
	**Expected** (Normal)	20(91)	22(100)	10	0.351	9(32)	19(68)	**111**	**0.007**	29(58)	41(82)	**41**	**0.032**
	**Mild** (Low roughness and elasticity)	1(5)	0(0)	−100		12(43)	8(29)	**−33**		13(26)	8(16)	**−38**	
	**Moderate** (Smooth, poor elasticity)	1(5)	0(0)	−100		5(18)	1(4)	**−80**		6(12)	1(2)	**−83**	
	**Severe** (Smooth, absent elasticity, loss of tonicity)	0(0)	0(0)	0		2(7)	0(0)	**−100**		2(4)	0(0)	**−100**	

**Table 4:** Mean pH ± standard deviation at T0 and T1.

Patient Group	Enrolled Patients	T0 Mean pH ± SD	T1 Mean pH ± SD
**Premenopausal (PMP)**	22	5.614 ± 0.899	5.409 ± 0.701
**Menopausal (MP)**	28	5.857 ± 0.621	6.125 ± 0.538

**Table 5:** The number of patients showing a change in vaginal pH, T1 vs. T0.

pH Change	Premenopausal PMP (n = 22)	Menopausal MP (n = 28)	Total (n = 50)
**Increase**	2	15	17
**Reduction**	7	7	14
**No variation**	13	6	19

**Table 6:** Amine presence (sniff test) and PMN score variation at T0 and T1 ∆% percentage change T1 vs. T0. Percentages are enclosed in parentheses, and statistically significant data (*p* < 0.05) are in bold.

**Amine Presence by Sniff Test**												
	**Premenopausal** **(PMP) ** **n = 22 (%)**				**Menopausal** **(MP)** **n = 28 (%)**				**Total** **n = 50 (%)**			
	T0	T1	**Δ%**	*p*	T0	T1	**Δ%**	*p*	T0	T1	**Δ%**	*p*
**Positive**	9(41)	5(23)	−44	0.195	7(25)	3(11)	−56	0.163	16(32)	8(16)	−50	0.061
**Negative**	13(59)	17(77)	30		21(75)	25(89)	19		34(68)	42(84)	23	
**PMN score**												
	**Premenopausal** **(PMP) ** **n = 22 (%)**				**Menopausal** **(MP)** **n = 28 (%)**				**Total** **n = 50 (%)**			
	T0	T1	**Δ%**	*p*	T0	T1	**Δ%**	*p*	T0	T1	**Δ%**	*p*
**Absent**	7(32)	5(23)	−28	0.631	2(7)	8(29)	>100%	0.450	9(18)	13(26)	44	0.611
**Rare or minimal**	10(46)	13(59)	28		22(79)	17(61)	−23		32(64)	30(60)	−6	
**Numerous**	4(18)	4(18)	0		4(14)	1(4)	−75		8(16)	5(10)	−37	
**Very numerous**	1(4)	0(0)	−100		0	2	/		1(2)	2(4)	100	

**Table 7:** In vitro isolated microorganisms at T0 and T1. Number of isolates and their distribution as monomicrobial or polymicrobial isolation for both study groups and total population.

	Premenopausal (PMP) n = 22		Menopausal (MP) n = 28		Total n = 50	
	T0	T1	T0	T1	T0	T1
**Isolated (n)**						
*Gardnerella vaginalis*	3	1	0	1	3	2
*Streptococcus agalactiae*	3	3	5	5	8	8
*Enterococcus faecalis*	2	2	6	3	8	5
*Staphylococcus aureus*	0	0	1	1	1	1
*Corynebacterium* spp.	1	1	0	0	1	1
*Escherichia coli*	1	4	6	5	7	9
*Citrobacter* spp.	0	1	0	1	0	2
*Klebsiella pneumoniae*	0	0	1	0	1	0
*Morganella morganii*	0	0	2	1	2	1
*Proteus mirabilis*	1	1	1	1	2	2
*Yeast*	2	1	1	1	3	2
**Total (n)**	13	14	23	19	36	33
**Isolation distribution (n)**						
*Monomicrobial*	8	4	6	6	14	10
*Polymicrobial*	3	5	9	7	12	12

**Table 8:** Ison–Hay Score and score variation at T0 and T1. The score variation was assessed by comparing the values before treatment (T0) and at follow-up (T1) according to the following criteria. Improvement: score reduction range from 4 to 1 and increase from 0 to 1 or 2; Worsening: score increase range from 1 to 4 and decrease from 2 to 0 or 1 to 0; No variation: same score at T0 and T1. Statistically significant data (*p* < 0.05) are in bold.

**Ison–Hay Score**										
		**Premenopausal** **(PMP)** **n = 22 (%)**			**Menopausal** **(MP)** **n = 28 (%)**			**Total** **n = 50 (%)**		
		T0	T1	*p*	T0	T1	*p*	T0	T1	*p*
Score	**Grade 0** No bacteria	4(18)	2(9)	**0.007**	13(46)	8(29)	0.063	17(34)	10(20)	**0.001**
	**Grade 1** *Lactobacillus-*dominated	7(32)	8(36)		2(7)	9(32)		9(18)	17(34)	
	**Grade 2** Lactobacilli and Gram- cocci	3(14)	12(55)		5(18)	8(28)		8(16)	20(40)	
	**Grade 3** Overgrowth of Gram- cocci and other morphotypes	5(22)	0(0)		7(25)	3(11)		12(24)	3(6)	
	**Grade 4** Gram+ cocci	3(14)	0(0)		1(4)	0(0)		4(8)	0(0)	
**Score variation**										
		**Premenopausal** **(PMP)** **n = 22 (%)**			**Menopausal** **(MP)** **n = 28 (%)**			**Total** **n = 50 (%)**		
**No variation**		11(39)			7(32)			18(36)		
**Improvement**		15(54)			12(54)			27(54)		
**Worsening**		2(7)			3(14)			5(10)		

**Table 9:** Patients selected for molecular analysis. Clinical and microbiological evaluation were categorized into three groups: ^a^ no variation (NV), ^b^ improvement (IMP), and ^c^ worsening (WR).

	**Premenopausal** **(PMP)** **n = 22 (%)**			**Menopausal** **(MP)** **n = 28 (%)**			**Total** **n = 50 (%)**		
**Patients selected for molecular analysis**	13 (59)			7 (25)			20 (40)		
	**Premenopausal** **(PMP)** **n = ** **13**			**Menopausal** **(MP)** **n = ** **7**			**Total** **n = ** **20**		
	NV ^a^	IMP ^b^	WR ^c^	NV ^a^	IMP ^b^	WR ^c^	NV ^a^	IMP ^b^	WR ^c^
**Variation after treatment**									
Symptoms of vaginal atrophy	5	8	0	2	5	0	7	13	0
Vaginal secretions	10	3	0	6	1	0	16	4	0
Vaginal mucosa color	11	2	0	3	4	0	14	6	0
Vaginal mucosa integrity	11	2	0	6	1	0	17	3	0
Vaginal mucosa thickness	11	2	0	3	4	0	14	6	0
Sniff test	7	5	1	4	1	2	11	6	3
pH	9	3	1	2	3	2	11	6	3
PMNs	7	4	2	3	4	0	10	8	2
Ison–Hay Score	2	10	1	0	7	0	2	17	1

**Table 10:** Differential DESeq2 analysis (T1 vs. T0) on OTUs belonging to bacterial genera related to biogenic amines (BA) in the PMP group. Changes in the bacteria are indicated via log fold change (logFC), while statistical significance is represented by *p*-values. Genera are listed in descending order according to the logFC, with negative values for T1 vs. T0 reduction and positive ones for increases. Statistically significant taxon differences are reported in bold.

PMP (n = 13), T1 vs. T0			
BA Producers’ Bacteria Genera (OTUs)	logFC	Dev.St.	*p*-Value
*Klebsiella* spp.	0.05	−0.02	0.907
*Clostridium* spp.	−0.16	−0.12	0.284
***Escherichia coli***	**−2.02**	**−5.90**	**<0.001**
*Mobiluncus* spp.	−1.61	−1.97	0.161
*Parvimonas* spp.	−1.92	0.24	0.200
*Leptotrichia* spp.	−1.93	−1.44	0.173
*Gardnerella* spp.	−2.58	−3.53	0.099
***Peptostreptococcus* spp.**	**−2.92**	**2.31**	**0.010**
*Megasphaera* spp.	−3.06	−2.96	0.136

**Table 11:** Differential DESeq2 analysis (T1 vs. T0) on OTUs belonging to bacterial genera related to biogenic amines (BA) in the MP group. Changes in the bacteria are indicated via log fold change (logFC), while statistical significance is represented by *p*-values. Genera are listed in ascending order according to the logFC, with negative values for T1 vs. T0 reduction and positive ones for increases. Statistically significant taxon differences are reported in bold.

MP (n = 7), T1 vs. T0			
BA producers Bacteria genera (OTUs)	logFC	Dev.St.	*p*-Value
***Escherichia coli***	**−6.78**	**−9.49**	**<0.001**
*Parvimonas* spp.	−1.15	−1.20	0.364
*Klebsiella* spp.	−0.08	−0.07	0.464
*Prevotella* spp.	0.06	1.91	0.748
*Enterobacter* spp.	0.09	−7.66	0.893
*Gardnerella* spp.	0.18	−0.59	0.927
*Clostridium* spp.	0.20	0.19	0.298
*Citrobacter* spp.	0.90	−16.00	0.457
***Peptostreptococcus*** **spp.**	**2.62**	**4.85**	**0.031**
***Mobiluncus*** **spp.**	**4.67**	**5.99**	**0.003**

## Data Availability

The data supporting this study are available on the Protocol.io platform (https://www.protocols.io/view/performance-and-safety-of-vaginal-administration-o-81wgbxmmqlpk/v1, accessed on 15 March 2024) and are also provided as a PDF file, identifiable with the following DOI: dx.doi.org/10.17504/protocols.io.81wgbxmmqlpk/v1.

## Supplementary Materials

The following supporting information can be downloaded at https://www.mdpi.com/article/10.3390/diseases12100237/s1, Table S1: Alpha-diversity; Table S2: Beta-diversity.
