# Supplementary material for: Retrospective Investigator-Initiated Trial on Tocopherol Acetate Vaginal Administration in Pre-and Postmenopausal Women
Source: Diseases. 2024 Oct 2;12(10):237. doi: 10.3390/diseases12100237 (PMC11506818; doi:10.3390/diseases12100237)
Supplement: Supplementary file 1 [file diseases-12-00237-s001.zip › diseases-3140137-supplementary.pdf]

Table S1. Alpha-diversity

Statistical comparison of the alpha diversity indexes between Pretreatment T0, treatment T1at taxonomic levels. The black values indicate the comparison was performed through the non paramatric test Mann-Whitney U test (p ≤0.05). The blue ones were calculated using the Whelch's t-test (p ≤0.05). Data set distribuition was checked by Shapiro-Wilk test.

	Chao1		Shannon	
	PMP	MP	PMP	MP
Taxon	T1vsT0	T1vsT0	T1vsT0	T1vsT0
Phylum	0.235	0.500	0.740	0.916
Class	0.137	0.793	0.504	0.430
Order	0.522	0.916	0.778	0.462
Family	0.228	0.027	0.317	0.401
Genus	0.293	0.916	0.343	0.529
Species	0.663	0.600	0.317	0.462

Premenopausal (PMP) Menopausal (MP)choorts
Pretreatment T0, treatment T1.

Tables S2 Beta-diversity

Tables show the distance matrix representing Bray-Curtis dissimilarity values between different samples. Each cell in the matrix contains the Bray-Curtis dissimilarity index for a pair of samples. This matrix is symmetric, with zeros along the diagonal because the dissimilarity between a sample and itself is always zero. Diagonal Elements: All are zero, indicating no dissimilarity within the same sample.Off-Diagonal Elements: These values range from 0 to 1. A value closer to 0 indicates high similarity between two samples, while a value closer to 1 indicates high dissimilarity.

Color Coding. The matrix is color-coded to visually represent the degree of dissimilarity: Green: High dissimilarity (values closer to 1) Yellow: Moderate dissimilarity Pink/Purple: Low dissimilarity (values closer to 0)

Sample code. PATIENTID_STUDYGROUP_TIME

Rows: Pretreatment T0; Colums: T1 Treatment.

High dissimilarity values for T1vsT0 are in bold.

Table S2A: Beta-diversity at species level for PMP cohort

	62_p_1	74_p_1	8_p_1	70_p_1	9_p_1	51_p_1	56_p_1	37_p_1	44_p_1	43_p_1	63_p_1	46_p_1	55_p_1
51_p_0	0.22	0.201	0.215	0.214	0.211	0.207	0.197	0.34	0.327	0.375	0.981	0.726	0.633
8_p_0	0.19	0.168	0.049	0.041	0.149	0.069	0.074	0.36	0.263	0.313	0.978	0.664	0.572
62_p_0	0.183	0.163	0.059	0.02	0.128	0.091	0.072	0.354	0.27	0.32	0.98	0.67	0.578
56_p_0	0.199	0.171	0.169	0.16	0.121	0.162	0.137	0.36	0.326	0.375	0.973	0.72	0.632
37_p_0	0.186	0.169	0.115	0.099	0.157	0.097	0.093	0.313	0.311	0.372	0.937	0.679	0.595
74_p_0	0.18	0.127	0.115	0.067	0.127	0.068	0.06	0.353	0.325	0.376	0.98	0.725	0.634
9_p_0	0.191	0.164	0.122	0.067	0.095	0.085	0.056	0.363	0.326	0.375	0.981	0.726	0.635
44_p_0	0.384	0.384	0.366	0.366	0.385	0.376	0.365	0.384	0.356	0.385	0.78	0.671	0.591
43_p_0	0.28	0.282	0.173	0.232	0.285	0.277	0.278	0.359	0.079	0.113	0.973	0.468	0.376

70_p_0	0.434	0.438	0.334	0.392	0.44	0.44	0.44	0.43	0.135	0.075	0.98	0.38	0.198
55_p_0	0.917	0.915	0.913	0.914	0.916	0.915	0.901	0.79	0.895	0.901	0.642	0.615	**0.857**
63_p_0	0.975	0.973	0.956	0.957	0.975	0.974	0.961	0.845	0.94	0.949	**0.665**	0.761	0.911
46_p_0	0.86	0.866	0.761	0.822	0.869	0.869	0.858	0.832	0.599	0.608	0.82	0.319	0.574

Table S2B: Beta-diversity at species level for MP cohort

	6_m_1	59_m_1	72_m_1	52_m_1	47_m_1	20_m_1	33_m_1
47_m_0	0.751	0.751	0.53	0.751	**0.722**	0.751	0.751
59_m_0	0.391	**0.238**	0.386	0.367	0.356	0.358	0.36
52_m_0	0.395	0.27	0.233	0.033	0.188	0.11	0.136
6_m_0	0.389	0.274	0.26	0.138	0.075	0.079	0.058
72_m_0	0.39	0.274	0.253	0.124	0.075	0.058	0.037
20_m_0	0.392	0.269	0.241	0.082	0.102	0.022	0.04
33_m_0	0.375	0.273	0.245	0.114	0.096	0.044	0.03
